# Electrophysiological and Neuroimaging Studies – During Resting State and Sensory Stimulation in Disorders of Consciousness: A Review

**DOI:** 10.3389/fnins.2020.555093

**Published:** 2020-09-15

**Authors:** Ritika Jain, Angarai Ganesan Ramakrishnan

**Affiliations:** Medical Intelligence and Language Engineering Laboratory, Department of Electrical Engineering, Indian Institute of Science, Bengaluru, India

**Keywords:** disorders of consciousness, sensory stimulation, EEG, fMRI, ERP, EMG, PET, resting-state analysis

## Abstract

A severe brain injury may lead to a disorder of consciousness (DOC) such as coma, vegetative state (VS), minimally conscious state (MCS) or locked-in syndrome (LIS). Till date, the diagnosis of DOC relies only on clinical evaluation or subjective scoring systems such as Glasgow coma scale, which fails to detect subtle changes and thereby results in diagnostic errors. The high rate of misdiagnosis and inability to predict the recovery of consciousness for DOC patients have created a huge research interest in the assessment of consciousness. Researchers have explored the use of various stimulation and neuroimaging techniques to improve the diagnosis. In this article, we present the important findings of resting-state as well as sensory stimulation methods and highlight the stimuli proven to be successful in the assessment of consciousness. Primarily, we review the literature based on (a) application/non-use of stimuli (i.e., sensory stimulation/resting state-based), (b) type of stimulation used (i.e., auditory, visual, tactile, olfactory, or mental-imagery), (c) electrophysiological signal used (EEG/ERP, fMRI, PET, EMG, SCL, or ECG). Among the sensory stimulation methods, auditory stimulation has been extensively used, since it is easier to conduct for these patients. Olfactory and tactile stimulation have been less explored and need further research. Emotionally charged stimuli such as subject’s own name or narratives in a familiar voice or subject’s own face/family pictures or music result in stronger responses than neutral stimuli. Studies based on resting state analysis have employed measures like complexity, power spectral features, entropy and functional connectivity patterns to distinguish between the VS and MCS patients. Resting-state EEG and fMRI are the state-of-the-art techniques and have a huge potential in predicting the recovery of coma patients. Further, EMG and mental-imagery based studies attempt to obtain volitional responses from the VS patients and thus could detect their command-following capability. This may provide an effective means to communicate with these patients. Recent studies have employed fMRI and PET to understand the brain-activation patterns corresponding to the mental imagery. This review promotes our knowledge about the techniques used for the diagnosis of patients with DOC and attempts to provide ideas for future research.

## Introduction

The word ‘consciousness’ has different connotations, depending upon the domain of discussion. In the clinical parlance, consciousness is described as consisting of two major components, namely arousal (eye-opening) and awareness (non-reflexive behavior or command following ability). Thus, clinical assessment of consciousness is primarily based on observations of the behavioral responses of a subject. Arousal is generally assessed by examining the presence of spontaneous or stimulus-induced eye opening, while the behavioral assessment of awareness relies on discriminating between automatic movements and response to commands or non-reflex actions (Guldenmund et al., 2012). Disorders of consciousness (DOC) is rather a broad term that includes several states with varying levels of consciousness, such as coma, vegetative state (VS)/unresponsive wakefulness syndrome (UWS), minimally conscious state (MCS), emergence from MCS (EMCS) and locked-in syndrome (LIS) (Giacino et al., 2002; Bodart et al., 2013). A severe injury to the brain (trauma, anoxia or stroke) may lead to coma, that might evolve into one of the above-mentioned clinical states. Coma is an acute state of unresponsiveness, without eye-opening or arousal (Gosseries et al., 2016). Patients in VS/UWS (Laureys et al., 2010) recover arousal systems, marked by the opening of eyes, but remain unresponsive to external stimuli and unaware of self and surroundings (Laureys and Boly, 2008). MCS patients may show some signs of consciousness (though fluctuating) by non-reflex behaviors. Patients in this state display limited, but clear evidence of self or surrounding awareness (Laureys et al., 2004). Recently, MCS is further categorized into two sub-states, MCS+ (higher-order signs of consciousness such as command-following) and MCS− (low-level signs of consciousness like visual pursuit of a salient stimulus or pain localization) (Bruno et al., 2011). LIS is a special case, in which the motor output of the patients is completely absent, but they have most of their cognitive functions preserved. Despite being behaviorally unresponsive, they display a level of consciousness similar to that of the healthy people (Bodart et al., 2013).

Various scales and scoring systems have been developed so far, such as Glasgow coma scale (GCS), disorders of consciousness scale (DOCS) or coma recovery scale-revised (CRS-R) (Giacino et al., 2004; Bodart et al., 2013; Reith et al., 2016). The primary features that are prominently considered in all these scoring systems are eye opening, verbal responses and motor responses. GCS is one of the most widely used score by the clinicians for detecting the level of consciousness (Reith et al., 2016). Though it is simple and easy to follow, it suffers from poor interrater reliability and also fails to distinguish between the VS/UWS and MCS states. GCS can only detect the relatively gross changes in the behavior and is unable to incorporate subtle changes, and thus may result in diagnostic errors (Schnakers, 2020). The misdiagnosis mainly occurs when the patients have covert awareness which fail to get captured by the behavioral assessment. These patients may possess motor deficits, sensory losses, language-impairment, fatigue or vigilance fluctuations and therefore, conventional bedside assessment based on clinical consensus or GCS scoring system have limited utility. The CRS-R is considered to be a reliable and standardized scoring system that was aimed to provide differential diagnosis of VS/UWS and MCS patients and thereby reduce the misdiagnosis (Guldenmund et al., 2012; Kondziella et al., 2020). Though CRS-R is extremely time-consuming and requires experienced personnel, it is considered to be the most reliable scale so far and true diagnosis is generally established through multiple assessments using the CRS-R scale (Kondziella et al., 2020).

Many studies have reported that about 37–43% of the patients diagnosed with VS/UWS showed signs of awareness (Andrews et al., 1996; Schnakers et al., 2009; Cruse et al., 2012; van Erp et al., 2015; Wade, 2018). van Erp et al. (2015) reported that 17 (39%) of 44 patients considered to be in VS/UWS turned out to be in MCS or were even conscious when examined with the CRS-R. Another study Schnakers et al. (2009) reported that 44 out of the 103 patients were diagnosed with VS based on the clinical consensus of the medical team. However, 18 (41%) out of those 44 were found to be in MCS following standardized assessment with the CRS-R. Andrews et al. (1996) also reported that 16 out of 40 (i.e., 40%) VS patients had some evidence of awareness. In many cases, patients who were previously assumed to be in vegetative state for many years have been able to communicate their thoughts using the neuroimaging methods, as shown by Fernandez-Espejo et al. (2008), Monti et al. (2010), and Cruse et al. (2011). All these studies clearly indicate that the reliability of diagnosis can be significantly improved using a more sensitive scale such as CRS-R and utilizing the neuroimaging techniques to detect the covert consciousness among these patients. A correct diagnosis of VS and MCS is of utmost importance, because it affects the treatment decisions and further therapeutic interventions. Fortunately, various neuroimaging techniques such as positron emission tomography (PET), functional magnetic resonance imaging (fMRI), electroencephalography (EEG) and transcranial magnetic stimulation (TMS) are being explored extensively to reduce the diagnostic errors and predict the recovery of consciousness. These tools have shown their potential by revealing signs of consciousness that are undetectable by the bedside clinical evaluation. Such promising results have motivated enormous research in the study of disorders of consciousness using neuroimaging techniques (Schnakers, 2020). These techniques may complement the clinical scoring systems, minimizing the risk of erroneous diagnosis.

Among various neuroimaging techniques, EEG is most widely used because it is non-invasive, portable, inexpensive, easy to set up and possesses high temporal resolution. Further, it can be easily used as a bedside-assessment tool for DOC patients. However, several studies (Boly et al., 2007; Di et al., 2007; Sharon et al., 2013; Liang et al., 2014; Bodien et al., 2017; Cheng et al., 2018) have also used fMRI, which provides both high-resolution structural imaging as well as functional imaging, thus giving crucial information for diagnosis that could possibly never be ascertained by EEG (Giacino et al., 2014). The biggest issue with fMRI is that it is extremely sensitive to movement artifacts and therefore is difficult to record for these patients.

This paper presents a review of various studies that have aimed to assess the level of consciousness of DOC patients, using different techniques including EEG, fMRI, PET, EMG (electromyography), EDA (electrodermal activity) or SCL (skin conductance level) and ECG (electrocardiogram), based on the resting-state or sensory stimulation methods. This review is an attempt to update our knowledge about the state-of-the-art methods used for the diagnosis and prediction of recovery from coma.

## Overview

**FIGURE 1:**
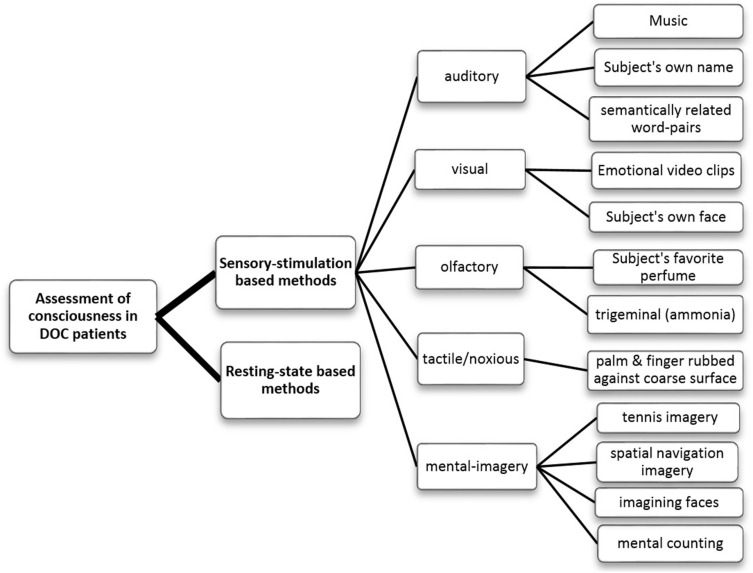
Flowchart categorizing the various methods of assessing the consciousness in DOC patients that have been reviewed in this paper.

In the literature, most of the studies related to the assessment of consciousness can be divided into two broad categories, namely, (a) sensory stimulation-based and (b) resting-state based analysis methods. As opposed to the sensory stimulation-based methods, resting-state analysis (Martinez et al., 2015; Schorr et al., 2016; Naro et al., 2018; Stefan et al., 2018; van den Brink et al., 2018; Cacciola et al., 2019; Wang et al., 2019) does not require the subjects to perform any specific task and it can provide valuable information about the spontaneous neural activity relevant to the fundamental brain state (Lv et al., 2018). On the other hand, sensory stimulation-based methods (Kotchoubey et al., 2005; Boly et al., 2007; Cruse et al., 2011; Schorr et al., 2015; Guger et al., 2017; Luauté et al., 2018; Schneider et al., 2018; Xiao et al., 2018; Agoiz Badia et al., 2019; Cacciola et al., 2019; Formisano et al., 2019; Huang et al., 2019; Xu et al., 2019) employ various modalities of sensory stimuli such as auditory, visual, olfactory, tactile and mental-imagery (Figure 1) and look for the expected cerebral response corresponding to the stimuli. These methods can help to study the integrity of the sensory pathways in the comatose patients. The paradigm used in sensory stimulation methods can be either active (Boly et al., 2007; Bekinschtein et al., 2008; Cruse et al., 2011; Schorr et al., 2015; Guger et al., 2017; Xiao et al., 2018; Huang et al., 2019; Xu et al., 2019) or passive (Mantini, 2007; Rossi Sebastiano et al., 2015; Li et al., 2018; Luauté et al., 2018; Schneider et al., 2018; Formisano et al., 2019; Portnova and Atanov, 2019). Active paradigms, such as mental imagery, require the attention or active participation of the subject, which may be challenging for the DOC patients due to their fluctuating vigilance levels and impaired cognitive functions. On the other hand, the passive paradigm is attention-independent and mostly aims to detect the brain’s automatic responses to the standard stimuli, as in the case of ERPs like mismatch negativity (MMN), N1, P2 or P3a (Strömmer et al., 2017).

**FIGURE 2:**
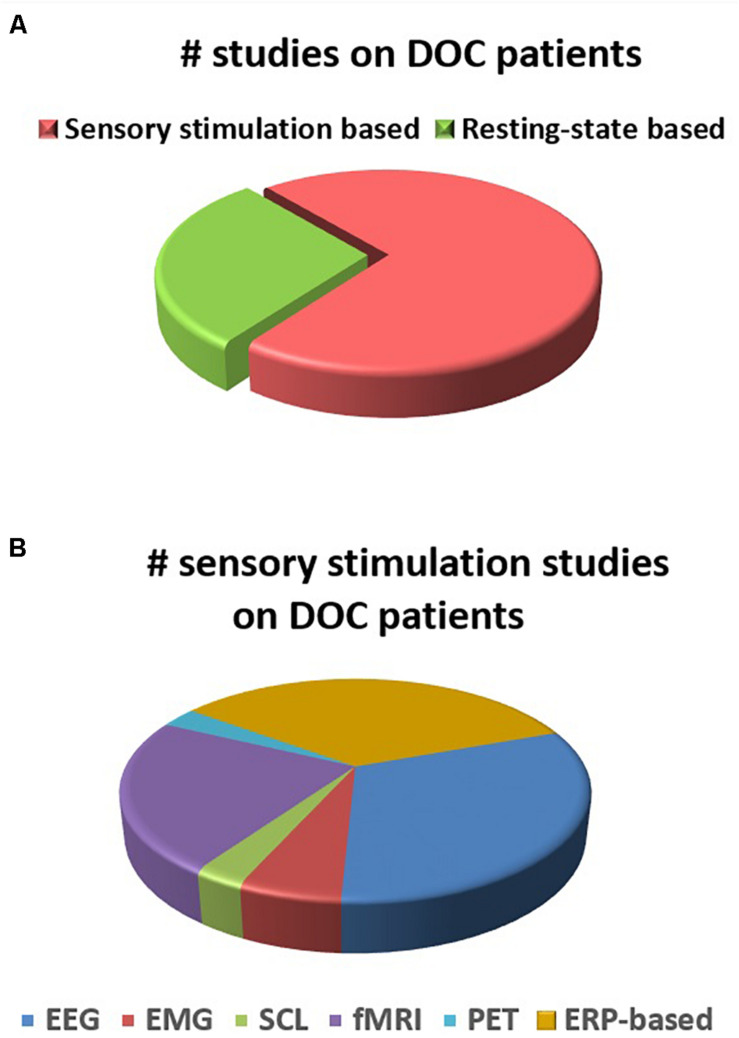
**(A)** Number of studies for DOC patients in sensory stimulation and resting state methods. **(B)** Number of studies within sensory stimulation using different electrophysiological and neuroimaging techniques.

**FIGURE 3:**
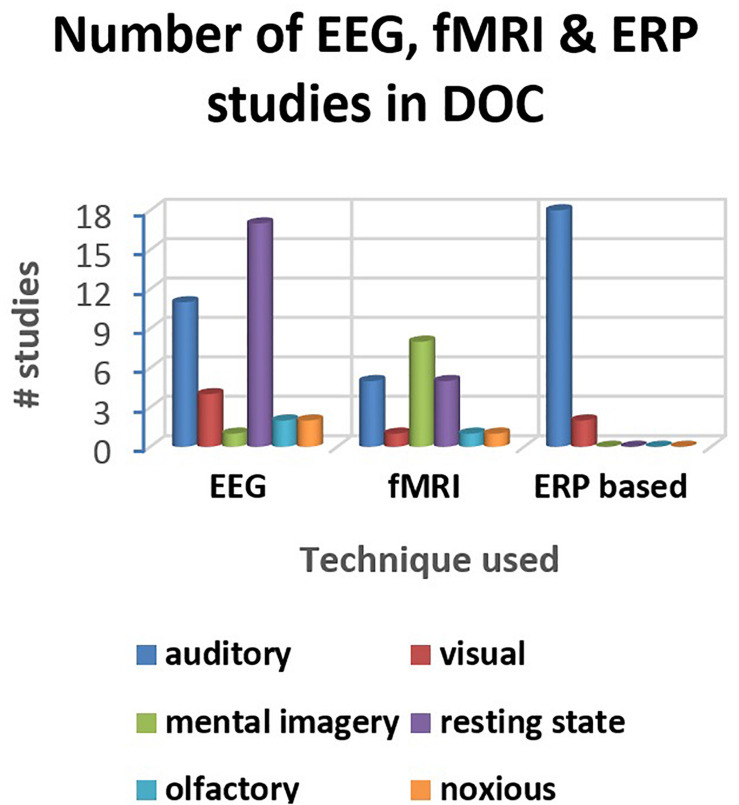
Distribution of the studies reviewed in the paper (for three major techniques- EEG, fMRI, and ERP).

A distribution of the number of studies (reviewed in this paper) using different electrophysiological and neuroimaging techniques is presented in Figures 2, 3. Figure 2A shows the dominance of sensory stimulation studies; however, resting-state methods have been recently explored and they have shown promising results for the diagnosis of DOC patients. Also, it is evident from Figure 2B that a majority of the sensory stimulation studies have used EEG and ERP for the assessment of consciousness in the patients with DOC.

More information about these studies are summarized in Supplementary Table S1.

## Studies Based on Sensory Stimulation

These methods employ different sensory stimuli and evaluate the corresponding brain responses. Based on the stimulus used, these can be sub-categorized into auditory, visual, tactile, olfactory or mental imagery (as shown in Figure 1). Further, emotion or some sense of familiarity attached to the stimuli has consistently been observed to evoke a much stronger response than the neutral stimuli lacking this aspect (Ninomiya et al., 1998; Bekinschtein et al., 2004; Di et al., 2007; Qin et al., 2010; Sharon et al., 2013; Castro et al., 2015; Luauté et al., 2018; Xiao et al., 2018; Huang et al., 2019). Many studies have therefore considered salient and emotionally charged stimuli such as subject’s own face (SOF) or familiar faces, emotional video clips, subject’s own name (SON) or narratives in a familiar voice (mother’s or children’s), or personal favorite music or perfume. Responses to the stimuli can be measured by various techniques (EEG, fMRI, PET, EMG, etc.), as mentioned earlier.

For the patients with DOC, EEG turns out to be the most suitable technique, since it can be easily deployed along the bedside of the patients. Event-related potentials (ERPs) are the most commonly reported EEG responses to tasks based on sensory stimulation (Ninomiya et al., 1998; Bekinschtein et al., 2004, 2009; Kotchoubey et al., 2005; Qin et al., 2008; Vanhaudenhuyse et al., 2010; Chennu et al., 2013; Sitt et al., 2014; Castro et al., 2015; Schorr et al., 2015; Beukema et al., 2016; Guger et al., 2017; Zhang et al., 2017; Kempny et al., 2018; Xiao et al., 2018; Agoiz Badia et al., 2019; Formisano et al., 2019). The reason behind the ERPs being so commonly used is that it is extremely difficult to obtain the small and subtle changes induced by sensory, motor or cognitive activities by simply using EEG, since these activities are buried in the noisy EEG signal (Vanhaudenhuyse et al., 2008). To get a handle of this issue, generally EEG responses to multiple repetitions of the stimulus are averaged. By doing so, the time-locked or event-related activity is revealed, which is referred to as ERP, while the spontaneous brain activity gets canceled out. ERPs reveal the time-course of information processing, starting from short-latency ERPs (time ranging from 0 to 100 ms from the onset of the stimulus) to mid-latency and long latency ERPs (order of several hundred ms). Short latency ERPs, such as somatosensory evoked potentials (SEPs) and brainstem auditory-evoked potentials (BAEPs) provide information only on the ascending pathways and not on the cognitive functioning and awareness of the person (Vanhaudenhuyse et al., 2008). The early/short-latency and mid-latency evoked potentials often reflect the brain’s automatic processing while the later ERP components, such as P300 and N400, are mainly associated with higher-order cognitive processing like stimulus discrimination, attention allocation or semantic and language processing (Davies et al., 2010).

Also, many studies (Bekinschtein et al., 2004; Perrin et al., 2006; Boly et al., 2007; Schnakers et al., 2008; Monti et al., 2013; Okumura et al., 2014) have investigated the role of fMRI in the evaluation of unresponsive patients, though it is difficult to perform fMRI of these patients.

### Studies Based on Auditory Stimulation

Among the different modalities in sensory stimulation, auditory modality has been extensively explored. Most of the studies (Bekinschtein et al., 2004, 2009; Di et al., 2007; Qin et al., 2008, 2010; Höller et al., 2011; Varotto et al., 2012; Chennu et al., 2013; Sitt et al., 2014; Castro et al., 2015; Schorr et al., 2015; Beukema et al., 2016; Henriques et al., 2016; Zhang et al., 2017; Kempny et al., 2018; Stefan et al., 2018; Agoiz Badia et al., 2019; Formisano et al., 2019; Portnova and Atanov, 2019) have used auditory stimulation, since it is much easier to conduct for the DOC patients, than visual and tactile stimulations.

#### Studies Based on Subject’s Own Name (SON)

A majority of the studies have used the subject’s own name (SON) as the auditory stimulus in contrast to others’ first names (OFN) or time-reversed OFN or simple sinusoid tones to understand the effect of familiarity or emotional saliency of the stimulus (O’Mahony et al., 1990; Perrin et al., 2006; Di et al., 2007; Fernandez-Espejo et al., 2008; Fischer et al., 2008, 2010; Qin et al., 2008; Schnakers et al., 2008; Bekinschtein et al., 2009; Fellinger et al., 2011; Castro et al., 2015; Kempny et al., 2018; Li et al., 2018; Stefan et al., 2018; Agoiz Badia et al., 2019).

##### Using EEG or ERP

In the literature, the most commonly reported ERP components, which have been extensively studied in DOC patients, are mismatch negativity (MMN), P300 and N400. Fischer et al. (1999) and Kane et al. (2000) found that mismatch negativity has a high specificity, but low sensitivity, and thus could not provide reliable prognostic information for the coma patients. However, Qin et al. (2008) could enhance the detection of MMN by using SON as the deviant stimuli. Hence, this SON-evoked MMN paradigm turned out to be very effective in evoking response in DOC patients, in contrast to the sinusoidal tones, which were used in MMN studies by Fischer et al. (1999) and Kotchoubey et al. (2013). The very first reports of P300 ERP responses in comatose patients by O’Mahony et al. (1990) suggested P300 to be linked to a favorable prognosis. Another early study (De Giorgio et al., 1993) further confirmed that the presence of P300 was significantly associated with the higher Glasgow coma scores (GCS) and awakening. However, P3 is elicited effectively only in attentive subjects, and sometimes can be absent even in normal subjects. So, to increase the probability of detecting P300 ERPs in coma patients, many researchers have used salient stimuli such as SON. Perrin et al. (2006) conducted an ERP study for VS, MCS and LIS patients to investigate the detection of SON among these groups. P3 component was observed in response to SON and not OFNs, among all the patients, except two out of five VS. This study has shown that the patients could detect salient stimuli, such as SON and hence possess partially preserved semantic processing capability. However, it cannot confirm the presence of conscious perception and awareness among the DOC patients.

Further, Schnakers et al. (2008) confirmed the results of Perrin’s study and added an active condition in the paradigm to check the voluntary brain processing in DOC. They found that MCS patients were able to voluntarily focus their attention on the targets, like that of controls, but none of the VS patients in this study showed any passive P3 response to SON. Another auditory ERP study by Fischer et al. (2010) was conducted to check if a robust MMN to duration-deviant tones and novelty P3 response to SON could be seen among the persistent vegetative state (PVS) and MCS patients. A novelty P3 component in response to SON could be seen in only 26% of the patients, unlike 80% of patients in a previous study by Perrin. This discrepancy might be due to different types of patients (PVS patients included in this study compared to VS) or different paradigms (SON as a novel stimulus among the standard and deviant tones in this study while SON as semantically different from seven other names in Perrin’s study).

A few studies (Holeckova et al., 2006; Keller et al., 2007) have shown the effect of familiar voice on SON stimulus; though they considered only healthy subjects. Holeckova et al. (2006) compared the effect of familiar voice versus unfamiliar voice on the response to SON stimulus in a passive oddball paradigm. This study has shown that even in the absence of attention, SON could elicit a larger response in the late phase of P300 and further larger parietal component (around 625 to 800 ms latency) for a familiar voice than for an unfamiliar voice. A similar study by Del Giudice et al. (2014) in both passive (just listen to SON or OFN by familiar or unfamiliar voice) and active condition (to count the target name) reported alpha desynchronization (i.e., event-related desynchronization or ERD) in the right parietal region in response to SON by familiar voice (FV) in the passive task. Along with alpha ERD, strong delta (event-related synchronization) ERS for the targets was also found in the active condition. This study further strengthened the role of SON uttered by a familiar voice as a novel stimulus that can be processed even without attention. Hence, it can be used for the DOC patients.

##### Using fMRI

Some researchers examined the effect of SON as auditory stimuli using fMRI. One such study by Di et al. (2007) showed that SON by familiar voice (SON-FV) induced activation in primary as well as higher-order associative temporal areas for all MCS and two VS patients (later recovered to MCS). Similar results were obtained by Wang et al. (2015) but in their study, they further investigated the relevance of etiology and prognostic value of SON-FV in the BOLD signal. They found that the activation type (lower level i.e., only primary auditory cortex or higher level i.e., beyond primary auditory cortex) and volume in the auditory cortex elicited by SON-FV significantly correlated with the prognosis of VS patients, particularly with traumatic etiology.

A case study by Bekinschtein et al. (2004) in an MCS patient demonstrated the activation of amygdala, insula and inferior frontal gyrus in response to the narratives presented in his mother’s voice in contrast to an unfamiliar voice. This study again hints toward the emotional and familiarity aspects of the stimulus, though it was carried out on only one patient and needs further validation.

#### Studies Based on Music

Some studies explored the effect of music on cognition in patients with disorders of consciousness.

##### Using EEG or ERP

Castro et al. (2015) showed that the response to SON was more often observed when the patient’s personal favorite music excerpt was played just before the stimulus, than the control condition consisting of a continuous music-like noise. This study suggests that music might have autobiographically primed the processing of SON. Further, all the patients who had a significant P3 ERP in music condition showed a favorable outcome while those without such a response failed to show a favorable outcome. This points to a possible link between the discriminative response to SON in music condition and the patient’s outcome. Thus, this study provides evidence for the beneficial effect of music on cognition in DOC patients.

To capture the neural correlates of emotion perception, Varotto et al. (2012) evaluated synchronization measures and EEG connectivity patterns induced by pleasant and unpleasant musical stimuli [36]. Among the five VS patients studied, only two of them (with less severe brain damage) showed changes in the network topology and increased connectivity for unpleasant music only. This contrasts with the observation for controls, where increased connectivity was found for pleasant music only. Although this study was carried out on a limited number of subjects, it has revealed that music stimulus has a role to play and can cause rearrangement of the brain networks among the VS patients.

A compelling and contradicting result by Portnova and Atanov (2019) showed that the EEG response to emotional stimuli in coma patients mostly reflects the physical parameters of stimuli and not its emotional content. Unlike the control group, in which EEG distances (in emotional space) correlated with emotional parameters (‘arousal’ and ‘pleasantness’) and not the physical parameters of sounds, coma patients showed a correlation of EEG distances with the acoustic parameters of the stimuli. Further, a correlation between EEG and loudness could be seen for the coma patients, which was absent for other groups. VS patients had emotional spaces closer to that of moderate TBI than coma patients. This suggests that while recovering from coma toward VS or MCS, their emotional processing improves. This result needs further validation by considering the neutral and emotional stimuli that are equalized in pitch and loudness.

Li et al. (2018) recently conducted an interesting study under three different stimulation types – music (a classical music excerpt), call-name (SON by relatives) and habit (wiping alcohol on lips for alcoholics or introducing smell of cigarette smoke for patients, who were smokers). It was found that EEG response under habit stimulation was higher than that for music, but lower than that for call-name stimulation. Also, such an effect was more pronounced for MCS than VS patients. This study reveals the effectiveness of habit stimulation. However, the detailed mechanism underlying habit remains unclear and needs further exploration.

##### Using fMRI

A study by Okumura et al. (2014) showed the effect of music stimulation in patients with diffuse brain injury using fMRI. They found that music stimulation resulted in the activation of bilateral superior temporal gyri (STG) in the control subjects, MCS patients as well as one VS patient, who later improved to MCS. This study suggested that the presence of STG activation may serve as an indicator of improvement in VS patients.

##### Using SCL

Luauté et al. (2018) used music and odor to elicit emotions and autobiographical memory of DOC patients. They analyzed the skin conductance level (SCL) during the preferred music and odor in contrast to neutral conditions but could not find any significant change between the conditions. On the other hand, Daltrozzo et al. (2010) have shown that a small but significant skin conductance response to emotional stimuli persists in some coma and low-responsive patients but drops as the level of consciousness decreases. These studies indicate that SCL may not be a reliable technique for the assessment of emotion or consciousness in coma patients.

#### Studies Based on Language Processing

To investigate the semantic and language processing in DOC patients, many studies have used N400 ERP, which is elicited by semantic deviance in the spoken language (generally unrelated word-pairs or sentences with anomalous endings). N400 ERP can serve as a measure to investigate if the linguistic functions are preserved in non-communicative patients (Rämä et al., 2010; Vanhaudenhuyse et al., 2010; Beukema et al., 2016; Formisano et al., 2019).

##### Using EEG or ERP

Steppacher et al. (2013) assessed information processing in 92 behaviorally unresponsive patients, diagnosed either as UWS (53) or MCS (39). They found a significant relation between the presence of N400 (but not P300) and subsequent recovery on follow-up. Also, more MCS than VS patients displayed N400, which was expected because MCS patients have comparatively stronger functional connectivity between the auditory cortex and a larger network of temporal and prefrontal cortices (Boly et al., 2004; Laureys et al., 2004).

Some studies have supported the idea that the absence of N400 ERP is significantly associated with the presence of aphasia diagnosed at the clinical follow-up (Hagoort et al., 1996; Swaab et al., 1997; Kielar et al., 2012; Formisano et al., 2019). N400 has shown higher reliability than P300 and MMN, and it indicates a positive or favorable outcome for VS and MCS patients (Vanhaudenhuyse et al., 2008; Steppacher et al., 2013; Formisano et al., 2019). However, the absence of N400 must be interpreted carefully; it might be due to aphasia and not necessarily implies poor outcome.

##### Using fMRI

Fernandez-Espejo et al. (2008) conducted an fMRI study to understand speech perception in VS and MCS patients after traumatic brain injury. They reported an increased cerebral response to language and complex sounds in some MCS as well as VS patients (who did not show any behavioral response to auditory stimuli or language comprehension). However, no relationship could be established between their fMRI responses and diagnosis of VS and MCS. This study has clearly proven that fMRI can play an important role in identifying the residual cognitive functions in DOC patients, which may remain undetected in a bedside examination.

Furthermore, a research study by Schiff et al. (2005) to investigate cortical responses to language and tactile stimulation in two MCS patients revealed that auditory stimulation with personalized narratives elicited activity in superior and middle temporal gyrus, similar to that of controls. However, for the time-reversed narratives, MCS patients failed to show response, while controls still showed activation. Also, for the tactile stimulation, both the MCS patients showed similar activation patterns as that of controls, except for the reduced volumes in the damaged hemisphere.

Hence, in auditory stimulation, responses to music and SON by a familiar voice have consistently implied good outcome of DOC patients. Although these ERPs – MMN, P300, and N400 indicate favorable outcome and serve as a good measure to predict recovery, they suffer from poor sensitivity. As a result, the presence of these higher-order cognitive ERPs indicates recovery but does not confirm that the patients are aware.

### Studies Based on Visual Stimulation

Visual stimulation has been less reported in DOC patients than the auditory stimulation. In visual stimulation, most of the studies have used stimuli such as subject’s own face (SOF) or a familiar photo or emotional video clips.

#### Using EEG or ERP

It is considered that the subject’s own face (SOF), just like SON, can serve as a salient visual stimulus. In line with this idea, a study by Ninomiya et al. (1998) evaluated the effect of familiarity on ERP latency and amplitude for the four visual stimuli namely subject’s own face (SOF), an unfamiliar face, a famous and familiar face and a red square among only healthy subjects. It was found that amplitudes and latencies of P300 in response to SOF were significantly larger and earlier, respectively, than those for non-target faces. However, this study did not include DOC patients.

For the bedside detection of awareness in DOC patients, Pan et al. (2014) developed a visual hybrid brain-computer interface (BCI) technique by combining P300 and steady-state visual evoked potential (SSVEP) responses. They considered the patient’s own photo and an unfamiliar photo as target stimuli. All but two patients (an MCS and a VS patient) failed to show any significant response. The proposed system could detect awareness in two out of seven DOC patients and could be used as a supportive tool for detecting consciousness in DOC patients.

A recent study by Huang et al. (2019) investigated the perception of emotions by presenting the patients with positive and negative emotional video clips. Though reliable responses were observed for three out of eight DOC patients, the activation pattern was not consistent among them. Further, the best response could be observed in an EMCS patient, followed by two MCS patients, while none of the VS patients could show activation.

#### Using fMRI

A case study of a traumatic DOC patient by Monti et al. (2013) assessed five passive, hierarchical levels of visual cognition, namely processing of light, color, motion, coherent shapes and object categories (faces and houses). At the active level, they also assessed the patient’s ability to voluntarily deploy visual attention on any one of two competing stimuli. Surprisingly, for all the visual hierarchy tests, the patient exhibited activations like that of controls. This suggests that not only visual processing for all the stages were intact, but also the patient was able to follow commands. However, this study was reported only for one patient and therefore needs to be validated on a larger sample.

Zhu et al. (2009) provided evidence of the residual functional substrates in MCS patients as the pictures of family members with emotional valence resulted in increased activation in the associated visual network. This observation goes in line with the idea that emotion and consciousness are closely related, and therefore a positive correlation could be seen between processing of emotions and the level of consciousness.

Many studies have reported that the emotional video clips and subject’s own face or any familiar face such as that of a friend/mother/spouse resulted in increased responses. However, the results are not consistent and indicate the need for further exploration.

### Studies Based on Olfactory Stimulation

It is considered that the sense of olfaction is functionally linked to emotional processes (Krusemark et al., 2013). Unlike other sensory systems, the olfactory system lacks an obligatory thalamic relay, which makes it unique with respect to both anatomical and functional organization (Price, 1985; Lundström et al., 2011). However, a high percentage of post brain injury patients suffer from anosmia (Sigurdardottir et al., 2010; Miao et al., 2015; Ilan et al., 2016; Nigri et al., 2016) and therefore, the olfactory stimulation has taken a back seat among the sensory stimulations that are used to assess the consciousness of these patients. As a result, very few studies have explored the effect of olfactory stimulation in DOC patients.

#### Using EEG or ERP

Schriever et al. (2017) performed a study to find the EEG power changes due to olfactory or trigeminal stimulation in healthy subjects and patients with olfactory impairment. By using continuous wavelet transform for time-frequency analysis, they found that the EEG power change was higher for olfactory stimulus (rose-like odor) than trigeminal stimulus (eucalyptol), and none for control stimulus (water) among the healthy subjects. Further, the EEG power change could reliably distinguish between the controls and patients with a high degree of accuracy.

Though this study did not consider the effect of olfactory stimulation on DOC patients, it has shown that the analysis of EEG power for an olfactory or trigeminal stimulation can provide some useful information.

#### Using fMRI

With an aim to explore the central olfactory processing in DOC patients, Nigri et al. (2016) conducted an fMRI study on a group of 26 VS and 7 MCS patients. This study revealed that a majority of VS (58%) and all the MCS patients demonstrated significantly preserved olfactory neural processing, and thereby showed activation in the primary olfactory region, i.e., piriform cortex. Interestingly, the extent to which the olfactory network was activated depended on the specific etiologies and not on the distinct diagnostic groups.

Further, they reported a significantly high activation within precuneus (commonly associated with the default mode network or DMN) for only DOC patients. According to their hypothesis, this increased precuneus activity shown by DOC patients could be a sign of failure to deactivate the DMN, unlike controls who are able to interrupt ongoing mental processes and deactivate DMN so as to focus on the given task. This study supports the notion that olfactory neural processing and cognitive reserve are preserved among these patients, particularly non-anoxic.

With the similar notion of functional coupling between olfactory and emotional processing, Sattin et al. (2019) tested 11 DOC patients. They used two assessment techniques, namely fMRI and the new olfactory discrimination protocol (ODP, comprising observation of behavioral responses like eyes closure, vocalization and head movement) for odor presentation and compared them. Though ODP is an easy and fast tool to be used along the bedside to test olfactory sense in DOC patients, it is not reliable (based on clinician’s score) and does not provide enough information for the ongoing neural processing. Still, this study suggests that the olfactory stimulation is rather an unexplored area and worth pursuing.

An interesting study by Pistoia et al. (2015) revealed covert abilities in an MCS patient through an innovative activation paradigm based on olfactory imagery. In this study, the patient was asked to imagine an unpleasant odor or to relax based on the visual stimulus (arrow pointing down and cross, respectively) shown on the screen. Surprisingly, it was found that the patient was able to activate, and rest purposefully, and also optimize his performance after a number of sessions. It shows that such a paradigm may be useful to detect covert signs of consciousness.

Heine et al. (2017) conducted a study to investigate the effect of auditory versus olfactory modality as well as the effect of preference (neutral versus preferred) in the test stimuli in 13 DOC patients. However, they assessed only behavioral responses of these patients, and did not analyze any electrophysiological signal. They found auditory stimuli, particularly preferred music, to be better than olfactory stimuli at enhancing arousal in these patients. This study further strengthens our previous conclusion that preferred music has the potential to improve the cognitive functions in DOC patients.

The olfactory system has an advantage that no widespread cortico-cortical and cortico-subcortical interactions are engaged to generate olfaction, unlike other complex sensory systems. This motivates further investigation and research in olfactory stimulation to study the covert signs of consciousness among patients with DOC.

### Studies Based on Tactile/Noxious Stimulation

Some research studies attempted to explore the effect of tactile stimulation (pain or touch) in patients with reduced levels of consciousness. These studies considered vibrotactile stimulators, massage on the left/right arm or stimulating the median nerve. To assess the implication of tactile stimulation on these patients, researchers have recorded EEG, EMG, SCL, heart rate (HR), PET, and fMRI.

#### Using EEG or ERP

A study by Venturella et al. (2019) showed an increased frontal and parietal activation in response to both touch and pain stimuli in VS patients. Out of the two, the pain-related stimulation resulted in greater activation and increased electrodermal and heart rate measures. Thus, the nociceptive stimulation seems to provide a consistent pattern and information about the covert responses.

Keller et al. (2007) studied the influence of tactile and auditory stimulation in PVS patients using multiple assessment techniques including EEG, EMG, SCL, and HR. For auditory stimulation, they considered white noise and a close relative’s voice and for tactile stimulation, they provided left or right arm massage. This study reported that non-specific acoustic stimulation (white noise) and tactile stimulation resulted in a significant increase in SCL as well as EMG activity. The greatest responses were obtained for the tactile stimulation, which also showed effect on HR and EEG activity. However, the result of this study seems contradictory to other studies that have reported an increased activation for the familiar voices. According to this study, a probable reason could be that these PVS patients were unable to process the semantic content of the stimuli delivered to them.

#### Using fMRI

Eickhoff et al. (2008) used fMRI to investigate the cortical responses to visual, auditory as well as tactile sensory stimulation on a TBI patient. They also studied the effect of speaker variability by presenting audio recordings from patient’s two children, two close female friends, and an unknown female student. All the recordings were such that the patient was directly addressed by the speaker (example: “hello X {mama/patient’s name}, this is Y {speaker’s name}). It was found that the visual stimulation only activated the left visual cortex, tactile stimulation of left forearm activated right primary (SI), secondary (SII) somatosensory areas and left cerebellum, while right-sided tactile stimulation showed bilateral SII activations. Also, stronger responses could be detected for speech directed to the patient and speaker-dependent modulation in the left amygdala and superior temporal sulcus. Interestingly, in both the regions, the children’s voices elicited the strongest activity, followed by friends’ and lastly the unknown voice. Further, addressing the patient resulted in larger activations than neutral (non-emotional) phrases, irrespective of the speaker. This result shows that though the patient is clinically unresponsive, without any sign of eye-opening, sleep pattern or even reaction to painful stimuli, he still shows cognitive and emotional speech processing capability.

#### Using PET

To grasp a better understanding of cerebral processing of noxious stimuli in DOC patients, Boly et al. (2008) conducted a PET study in 5 MCS and 15 PVS patients. They studied brain activation induced by bilateral electrical stimulation of the median nerve in these patients. Noxious stimulation activated the whole cortical pain matrix (S1-primary somatosensory cortex, thalamus, insular, frontoparietal and anterior cingulate cortices) in both MCS and controls in a similar fashion. In contrast, PVS patients only showed activation in the contralateral thalamus and S1. Further, MCS patients had preserved functional connectivity between S1 and cortical network that includes frontoparietal associative cortices, unlike PVS patients.

A similar study was conducted by Laureys et al. (2002) using high-intensity electrical stimulation of the median nerve in PVS patients using PET. This study also reported increased neuronal activity in the primary somatosensory cortex, though the activation was isolated in all PVS patients, even if the resting brain metabolism was severely impaired. Also, the functional connectivity analysis showed that the observed activation in primary sensory cortical area existed as an island with a lack of association with higher-order cortices, considered to be necessary for the presence of awareness. This idea of a “missing link” between the primary and higher-order associative areas in the brain being responsible for the lack of awareness and hence consciousness, has been supported by many studies.

Neuroimaging studies have shown that the level of activation of the anterior cingulate cortex (ACC) correlates with pain intensity scores. The activation of ACC in MCS patients, as found in a study by Derbyshire et al. (1997) indicates that they might have pain affect similar to that of controls. This study therefore provides evidence of potential pain perception capacity in MCS patients.

### Studies Based on Mental-Imagery Stimulation

Some studies tried to test communication in DOC patients using mental imagery by evaluating their EEG or fMRI activation patterns (Bekinschtein et al., 2004; Boly et al., 2007; Monti et al., 2010; Cruse et al., 2011; Sharon et al., 2013; Liang et al., 2014; Bodien et al., 2017).

#### Using EEG or ERP

Studies based on mental imagery tasks have mostly used fMRI, only a handful of studies (Cruse et al., 2011; Goldfine et al., 2011) have considered using EEG. Cruse et al. (2011) aimed to assess awareness among VS patients using EEG. They found that 3 out of 16 VS patients could reliably and consistently generate appropriate EEG responses to both the motor imagery tasks (“right hand squeezing into a fist” and “toes wiggling”), despite being behaviorally unresponsive.

Goldfine et al. (2011) used EEG power spectral analysis to determine awareness among 3 brain-injured patients (2 MCS and 1 LIS). This study considered motor imagery (“imagine swimming”) and spatial imagery (“imagine walking through your house”) and reported significant changes in EEG power spectra among 2 patients (1 LIS and 1 MCS). Though the changes were inconsistent, they provided evidence of command following. This indicates that EEG power spectral analysis could be a usable awareness detection tool at the bedside. However, it needs further validation by considering a larger sample size (including VS patients as well, which were not considered in this study).

#### Using fMRI

Liang et al. (2014) considered four such imagery tasks (imagine navigating home, imagine playing tennis, imagine familiar faces, mental counting and rest) and found that different tasks activated the brain differently, with the strongest activation in the parahippocampal and premotor area for the “navigation” task, and in superior parietal cortex as well as premotor area for “playing tennis” task in the controls. Some patients showed activations similar to that of controls for the navigation and counting tasks. These results are consistent with Boly et al. (2007), who showed that “navigation” and “playing tennis” produced the most distinct activation patterns compared to “imagine faces” and “mental rehearsal of song.” Further, Liang’s study reported that in addition to “navigating home” and “playing tennis,” mental calculation is also a robust mental imagery task.

Another fMRI study on a similar track by Monti et al. (2010) successfully applied a simple question-answer task using mental imagery on a DOC patient, who showed reliable responses during both the imagery (spatial navigation and motor imagery) tasks. Along with this patient, four other (out of 54) patients could also willfully modulate their brain activities and showed reliable activation in at least one of the two imagery tasks. Further, Bodien et al. (2017) showed that the healthy subjects performed better in tennis imagery task, while in DOC patients, hand squeezing motor imagery task detected command following with higher accuracy than tennis imagery.

All these studies have shown that a small proportion of VS or MCS patients have some awareness and cognition, which remains undetected even by a careful clinical examination. Hence, the EEG and fMRI techniques can be extremely useful in motor imagery paradigms to detect the covert signs of awareness and further establish basic communication with such patients, who seem to be unresponsive.

Some studies (Bekinschtein et al., 2008; Habbal et al., 2014; Lesenfants et al., 2016) have also used EMG to detect the responses to command in DOC patients, since some micromovements might go undetected while using standard diagnostic scales. Bekinschtein et al. (2008) found a significant increase of EMG signal in a VS patient in response to a target command (“Move your hand”) as compared to the control command (“Today is a sunny day”). Along the same line, Habbal et al. (2014) conducted an EMG based study with a comparatively larger sample (10 VS, 8 MCS−, 20 MCS+) and three different target commands (“Move your hands,” “Move your legs,” and “Clench your teeth”). They found that the command “Move your hand” produced the most frequent responses in both the controls (83% of cases) and patients (three out of 4 unresponsive patients). A response was obtained for at least one of the target commands in 6 VS, 3 MCS− and 11 MCS+ patients. Hence, this technique can serve as an objective measure to detect volitional responses in DOC patients. Lesenfants et al. (2016) reported that EMG responses were detected in all the MCS+, EMCS, and LIS patients, but none of the VS patients. This study again proves the potential use of EMG as an objective bedside evaluation tool. Further, EMG is the most convenient, easy, portable and inexpensive among all the neuroimaging and electrophysiological techniques.

Rossi Sebastiano et al. (2015) conducted a study to verify the value of multiple neurophysiological tests in the classification of DOC patients. It included overnight sleep recordings of EEG, ECG, EOG, and EMG in a large patient population. Their findings suggest that a combination of measures like multimodal evoked potential and EEG can give significant information about the residual functions in these patients.

Furthermore, to understand the non-linear dynamics of the brain, some studies have utilized connectivity and complexity measures. King et al. (2013) designed a novel measure called weighted symbolic mutual information (wSMI) to quantify information transfer across distant cortical and thalamic areas. They reported that wSMI is a robust and good enough measure that can index the state of consciousness, minimize common-source artifacts and improve discriminability. Also, it outperformed the power spectrum measures in discriminating VS from MCS patients.

Sitt et al. (2014) conducted a high-density EEG study among a large sample of 167 DOC patients, using auditory stimulation (‘Local-Global’ paradigm) and evaluated a series of EEG-derived measures [early ERP components such as P1, MMN, and CNV, late ERP components (P3a and P3b), power in frequency bands, spectral entropy, permutation entropy, complexity, phase lag index, imaginary coherence, and wSMI]. They found low-frequency power, EEG complexity and information exchange to be the most reliable signatures of the conscious state. However, as per the literature, most of the studies involving the complexity and connectivity analysis are based on the resting-state activity of the brain.

## Studies Based on Resting-State Analysis

Instead of recording the electrophysiological signals while providing sensory stimulation, researchers are exploring resting-state activity, which can provide valuable information about the intrinsic brain activity (stimulus-independent). Resting-state fMRI is the most popular and state-of-the-art technique to investigate the functional architecture of the brain and various resting-state networks (RSNs including DMN). However, clinical applications of this technique are still at an early stage of development (Ilan et al., 2016). MR scanners turn out to be uncomfortable for the DOC patients. Also, they need to be sedated in order to minimize undesired movements in the scanner, which in turn affects the study being conducted (Lechinger et al., 2013). Hence, resting EEG is used as an alternative, especially for DOC patients, as it allows for some movement and can be easily applied at the bedside.

Features derived from these resting-state studies can be extremely useful to monitor the brain conditions of DOC patients. Feature-based approaches can be broadly grouped as (a) power spectral analysis (b) complexity analysis [Lempel-Ziv complexity, approximate entropy (ApEn), cross-entropy, permutation entropy (PE), etc.] (c) connectivity analysis (phase locking index, partial directed coherence, wSMI, imaginary part of coherence, Granger-causality, mutual information, etc.).

Some of the earlier studies measured spectrum powers and reported that VS patients showed increased delta power, but decreased alpha power compared to MCS patients. The patient group showed higher delta but lower alpha and beta power than controls (Lehembre et al., 2012; Lechinger et al., 2013; Schorr et al., 2016; Naro et al., 2018). Also, power ratio index (PRI), i.e., ratio of power in the delta and theta frequency bands (slow-wave activity) to that in the alpha and beta frequency bands (fast-wave activity) showed a negative correlation with CRS-R scores of patients. Further, Coleman et al. (2005) reported a significant relationship between neuronal electrical activity and regional glucose metabolism in all MCS patients, but in none of the VS patients (indicating impaired coupling between neuronal electrical function and cerebral energy metabolism).

Non-linear analysis of resting EEG using indices like complexity and entropy has also been used in several studies (Sarà and Pistoia, 2010; Gosseries et al., 2011; Sarà et al., 2011; Wu et al., 2011; Sitt et al., 2014; Stefan et al., 2018; Zhu et al., 2019) to quantify the degree of consciousness in DOC patients. Entropy of EEG is a measure of its regularity. Thus, a higher value of entropy indicates that the subject is awake, while lower values indicate deeper unconsciousness (Bruhn et al., 2003; Gosseries et al., 2011; Wu et al., 2011; Thul et al., 2016). It has been found that the mean values of approximate entropy (ApEn) were significantly lower in patients than controls (Sarà and Pistoia, 2010; Sarà et al., 2011; Wu et al., 2011; Stefan et al., 2018) and also the mean EEG entropy values for DOC patients had a positive linear correlation with CRS-R scores (Bruhn et al., 2003). Further, Lempel-Ziv complexity (LZC) and cross-approximate entropy values were significantly lower for the PVS, followed by MCS, and the highest for controls (Wu et al., 2011).

Functional connectivity has also been employed to assess the level of integration and connection of brain networks (Vanhaudenhuyse et al., 2010; Lehembre et al., 2012; Höller et al., 2014; Sitt et al., 2014; Schorr et al., 2016; Chennu et al., 2017). The most commonly used measure of connectivity is coherence (C), but it can be contaminated by volume conduction or artifactual correlation between the electrodes (Nunez et al., 1997; Srinivasan et al., 1998; Lehembre et al., 2012; Schorr et al., 2016; Stefan et al., 2018). Other approaches such as phase locking index, phase coherence, imaginary part of coherence (IC) and phase lag index (PLI) have been used very frequently in most of the studies. Lehembre et al. (2012) compared all the three methods (C, IC, and PLI) in DOC patients and found higher frontal-to-posterior connectivity in MCS than VS patients using PLI and IC; however, coherence failed to provide any information. A study by Höller et al. (2013) considered 44 features for EEG connectivity analysis and three, namely partial coherence, directed transfer function and generalized partial directed coherence, out of these 44 features could distinguish between MCS and VS patients at above-chance accuracy levels for all the 49 patients.

Stefan et al. (2018) applied various measures such as microstates, approximate entropy, power in alpha and beta bands, wSMI, symbolic transfer entropy and complex network analysis to index consciousness and predict outcome in DOC patients using high-density EEG. This study aimed to develop an automated system by selecting an optimal subset of features that can be used for the classification of two categories of outcome: (i) VS or dead, and (ii) MCS or EMCS, in severe DOC patients. It obtained a high prediction power by combining the following three metrics: microstate with topography right-frontal to left-posterior in the 2–20 Hz frequency band, path length and clustering coefficient obtained from thresholding alpha coherence. Also, they reported an increase in low-frequency oscillations in VS patients, which is in line with other studies (Lehembre et al., 2012; Schorr et al., 2016; Naro et al., 2018) (alpha and beta power were significantly lower in VS than MCS patients, while low frequency delta power showed the opposite pattern). Soddu et al. (2012) aimed to identify the default-mode network (DMN) in DOC patients using spatial independent component analysis. They found fewer connections in default-mode areas in VS patients, compared to the controls as well as LIS patients. Ovadia-Caro et al. (2012) studied the resting state inter-hemispheric connectivity in externally oriented network areas, and not in DMN, among DOC patients. They reported reduced connectivity in subjects with impaired awareness compared to those with intact awareness, indicating a positive correlation between the functional connectivity and the level of consciousness.

## Discussion

Disorders of consciousness encompass states with varying levels of impaired consciousness, and it is difficult to draw a distinction between these states (VS, MCS+, MCS−, and EMCS), especially by relying only on the behavioral evaluation. Making a correct diagnosis is extremely important since it affects the prognosis and further treatment therapies of DOC patients. The neuroimaging techniques can play an important role to glean the covert cognitive capabilities of the behaviorally unresponsive patients and thereby reduce the misdiagnosis rate among these patients.

With an aim to improve the diagnosis, researchers have attempted various active or passive sensory stimulation methods. Most of the sensory stimulation studies in the literature converge to the conclusion that emotional or autobiographically salient stimuli like SON or SON-FV, SOF or family pictures, narratives in familiar voices and music evoke stronger responses than the neutral or non-salient stimuli such as sinusoidal tones.

Not only the choice of stimuli, but also the analysis technique being used to study the corresponding responses are crucial for the assessment of consciousness. Among the analysis techniques, EEG, fMRI, and ERP are the most widely adopted (as is evident from Figures 2B, 3). EEG is much easier to conduct for these patients than fMRI, as the latter is noisy, non-portable and requires minimal or restricted movement of the subject. Most ERP based studies have evaluated N100, MMN, P300, and N400 responses to passive paradigms in order to investigate the intactness of sensory pathways, pre-attentive automatic discrimination, active information processing, semantic, speech and language processing in patients with impaired consciousness. Still, the presence of these ERPs does not provide complete information about the level of awareness in these patients. On the other hand, active paradigms such as mental imagery can confirm the conscious perception and awareness among the DOC patients. But it might be difficult for the DOC patients to perform mental imagery tasks. Therefore, the inability of a patient to perform a mental imagery task does not imply the absence of awareness in the patient. Considering all the ERP-based studies, we can reach a conjecture that MMN, P300, and N400 are all predictors of a favorable outcome, but the absence of these ERPs needs to be interpreted carefully.

Some studies have also shown the use of EMG as a potent bedside evaluation tool, which can provide valuable information to detect the volitional responses in DOC patients. Moreover, EMG has a great advantage of being extremely convenient and inexpensive.

Recent studies have shown a huge interest in resting-state EEG and fMRI analysis, since it is independent of the patient’s ability to understand the instructions. These studies have provided enough evidence of reduced DMN and inter-hemispheric connectivity as well as reduced entropy and other complexity measures in VS patients. Reviewing all the resting state-based studies, we found that spectral power measures and coherence in the alpha frequency band are highly correlated with the level of consciousness, i.e., high alpha power and alpha coherence provide evidence of recovery with high predictive sensitivity and specificity. In addition, the connectivity analysis of resting-state EEG using measures such as wSMI, dwPLI and IC can help distinguish between different conscious states (VS, MCS, and EMCS).

## Conclusion

To conclude, sensory stimulation-based methods have shown a huge promise to detect the covert consciousness in DOC patients, especially with the choice of salient and emotionally charged stimuli. In terms of the modality of sensory stimulation, auditory stimulation has been the dominant one, adopted by the majority of the studies. Other modalities like tactile and olfactory have been less explored and have a lot of scope for further research. Also, the resting state analysis methods using connectivity, entropy and complexity measures have shown tremendous value for the assessment of consciousness in coma. So, both the methods are potent in improving the diagnosis as well as prediction of recovery in comatose patients. The techniques like EEG and fMRI can provide valuable information for identifying the residual cognitive capabilities in the DOC patients, which remain undetected (or uncovered) in a clinical evaluation resulting in misdiagnosis and incorrect treatment therapy. Also, EMG seems to be a promising option to detect the ability of command-following in these patients. EEG and EMG are portable, cheap, and convenient to set-up and therefore can be easily incorporated in the ICU and clinical settings.

In our opinion, multimodal sensory stimulation involving emotionally charged stimuli tailored specifically for each patient, together with functional connectivity measures evaluated in the resting state using EEG can potentially distinguish VS/UWS from MCS. Further, it may assist to improve the prognosis and thereby, prediction of recovery for the DOC patients.

## Author Contributions

AR and JR contributed to the conception and design of the study. JR reviewed the literature and wrote the first draft.

## Conflict of Interest

The authors declare that the research was conducted in the absence of any commercial or financial relationships that could be construed as a potential conflict of interest.

## Supplementary Material

TABLE S1 A comprehensive review of the literature studied in this article.
